# Pulse wave velocity in real-time cardiac magnetic resonance

**DOI:** 10.1186/1532-429X-16-S1-P382

**Published:** 2014-01-16

**Authors:** Martin Fasshauer, Johannes T Kowallick, Arun Joseph, Christina Unterberg-Buchwald, Klaus-Dietmar Merboldt, Michael Steinmetz, Jan M Sohns, Wieland Staab, Andreas Schuster, Dirk Voit, Sebastian Schaetz, Shoun Zhang, Jens Frahm, Joachim Lotz

**Affiliations:** 1 Institute for Diagnostic and Interventional Radiology, University Medical Center Göttingen, Göttingen, Germany; 2 Biomedizinische NMR Forschungs GmbH, Max-Planck-Institut für biophysikalische Chemie, Göttingen, Germany; 3 Clinic for Cardiology and Pneumology, Heart Center, University Medical Center Göttingen, Göttingen, Germany; 4 Clinic for Pediatric Cardiology and Intensive Care Medicine, University Medical Center Göttingen, Göttingen, Germany; 5 partner site Göttingen, DZHK (German Cardiovascular Research Center), Göttingen, Germany

## Background

Atherosclerosis and its associated diseases are constantly increasing in developed countries. Aortic stiffness is an indicator for atherosclerosis and is associated with mortality and morbidity especially in aortic abdominal aneurysms. For further evaluation of aortic stiffness, we examined n = 13 healthy volunteers using real-time magnetic-resonance imaging (RT-MRI) with highly undersampled radial fast low-angle shot (FLASH) acquisitions, phase-sensitive image reconstructions and regularized nonlinear inversion (NLINV). We hypothesized that RT-MRI is able to determine pulse wave velocity (PWV) and flow data using just one transverse view of the ascending and descending aorta. This method could be superior to complex known MRI methods using velocity projection today.

## Methods

We assessed PWV as surrogate parameter for aortic stiffness by velocity-encoded RT-MRI. Time lag between the ascending and descending aortic pulse wave was calculated and divided by the mean length of the aortic arch for each individual in detail. RT-MRI can determine PWV during normal breathing, physical strain and recovery from strain using the Valsalva (VM) and Mueller maneuvers (MM). During strain/maneuvers volunteers had visual feedback of intra-thoracic pressure via a mouthpiece to ensure adequate strain performance. We calculated PWV in normal breathing, at the end of strain and at the end of the recovery phase. Due to the advantage of a single heart beat-to-beat variability with RT-MRI, the average PWV of 4 heartbeats could be calculated in each phase with Standard deviation (SD).

## Results

While changes during MM were very subtle (normal breathing 3.23 m/s ± 0.05 SD vs. MM 2.99 m/s ± 0.04 SD vs. recovery 3.24 m/s ± 0.05), increase and especially decrease during recovery from VM was more pronounced (normal breathing 3.55 m/s ± 0.06 vs. VM 4.12 m/s ± 0.08 vs. recovery 3.13 m/s ± 0.06, VM vs. recovery p < 0.05, values shown as mean ± SEM).

## Conclusions

Successful measurements of PWV in one transverse view during physical strain have not been shown before to our knowledge and prove to be an easy alternative to previous MR attempts determining PWV. In addition, the significant PWV decrease measured in RT-MRI from VM to recovery resembles the normal variability of aortic stiffness in healthy subjects due to change in intra-thoracic pressure. Real time MRI is able to measure these data under stress maneuvers such as the VM or MM. A combined score of PWV and its variability might be helpful for risk assessment in the development of aortic aneurysms

## Funding

This work was funded by the Deutsche Forschungsgemeinschaft (LO 1773/1-1).

